# Identification of a m^6^A-related ferroptosis signature as a potential predictive biomarker for lung adenocarcinoma

**DOI:** 10.1186/s12890-023-02410-x

**Published:** 2023-04-18

**Authors:** Dongdong Li, Ting Chen, Qiu-Gen Li

**Affiliations:** 1 Medical College of Nanchang University, Nanchang, 330006 Jiangxi P. R. China; 2 Department of Pulmonary and Critical Care Medicine, Jiangxi Provincial People’s Hospital, The First Affiliated Hospital of Nanchang Medical College, Nanchang, 330006 Jiangxi P. R. China; 3 Department of Pulmonary and Critical Care Medicine, Wuhan Wuchang Hospital, Wuhan, 430063 Hubei P. R. China

**Keywords:** Bioinformatics, Ferroptosis, Lung adenocarcinoma, m^6^A, Prognosis

## Abstract

**Background:**

Both N6-methyladenosine (m^6^A) and ferroptosis-related genes are associated with the prognosis of lung adenocarcinoma. However, the predictive value of m^6^A-related ferroptosis genes remains unclear. Here, we aimed to identify the prognostic value of m^6^A-related ferroptosis genes in lung adenocarcinoma.

**Methods:**

Lung adenocarcinoma sample data were downloaded from the University of California Santa Cruz Xena and Gene Expression Omnibus databases. Spearman’s correlation analysis was used to screen for m^6^A-related ferroptosis genes. Univariate Cox regression, Kaplan–Meier, and Lasso analyses were conducted to identify prognostic m^6^A-related ferroptosis genes, and stepwise regression was used to construct a prognostic gene signature. The predictive value of the gene signature was assessed using a multivariate Cox analysis. In the validation cohort, survival analysis was performed to verify gene signature stability. The training cohort was divided into high- and low-risk groups according to the median risk score to assess differences between the two groups in terms of gene set variation analysis, somatic mutations, and tumor immune infiltration cells.

**Results:**

Six m^6^A-related ferroptosis genes were used to construct a gene signature in the training cohort and a multivariate Cox analysis was conducted to determine the independent prognostic value of these genes in lung adenocarcinoma. In the validation cohort, Kaplan–Meier and receiver operating characteristic analyses confirmed the strong predictive power of this signature for the prognosis of lung adenocarcinoma. Gene set variation analysis showed that the low-risk group was mainly related to immunity, and the high-risk group was mainly related to DNA replication. Somatic mutation analysis revealed that the TP53 gene had the highest mutation rate in the high-risk group. Tumor immune infiltration cell analysis showed that the low-risk group had higher levels of resting CD4 memory T cells and lower levels of M0 macrophages.

**Conclusion:**

Our study identified a novel m^6^A-related ferroptosis-associated six-gene signature (comprising SLC2A1, HERPUD1, EIF2S1, ACSL3, NCOA4, and CISD1) for predicting lung adenocarcinoma prognosis, yielding a useful prognostic biomarker and potential therapeutic target.

**Supplementary Information:**

The online version contains supplementary material available at 10.1186/s12890-023-02410-x.

## Background

Lung cancer is one of the most common cancers (accounting for 11.6% of all cancer diagnoses) and the leading cause of cancer-related deaths worldwide (18.4% of total cancer mortality), with an approximate 2.2 million new cases and 1.79 million deaths per year [1, 2]. Lung adenocarcinoma (LUAD) is the most common type of lung cancer, accounting for approximately 40% of cases [3]. Although comprehensive therapies such as chemotherapy, radiation therapy, and molecular targeted therapy have provided advanced LUAD treatment options, the 5-year survival rate remains only 15% [4, 5]. Therefore, identifying useful diagnostic, therapeutic, and prognostic markers is an urgent goal.

N6-methyladenosine (m^6^A), the most abundant RNA modification in eukaryotic cells, plays an important role in various biological processes and mRNA metabolism by regulating translation, processing, stabilization, and degradation of the target RNA [6–8]. m^6^A has been associated with various cancers, such as colorectal cancer, adrenocortical carcinoma, bladder cancer, and lung cancer [3, 9–11]. Zhuang et al. constructed a robust diagnostic model using 11 m^6^A molecules and a prognostic model using 10 m^6^A molecules for LUAD [12]. Yin et al. reported that m^6^A RNA methylation-mediated RMRP stabilization promotes non-small-cell lung cancer (NSCLC) progression by regulating the TGFBR1/SMAD2/SMAD3 pathway [13]. In addition, Li et al. found that the m^6^A reader YTHDF2 contributes to LUAD progression by targeting AXIN1/Wnt/β-catenin signaling [14].

Ferroptosis is a non-apoptotic type of regulated cell death that is associated with oxidative damage [15] and characterized by an iron-dependent accumulation of lipid peroxidation and subsequent damage to the plasma membrane [16]. Previous studies have shown that certain genes can drive ferroptosis, whereas others can negatively regulate ferroptosis [17, 18]. Ferroptosis-related genes may be promising therapeutic targets for anticancer drug research and cancer treatment [19]. Researchers have also identified a potential link between m^6^A molecules and ferroptosis genes in tumor development [20, 21]. The m^6^A reader YTHDC2 is a powerful endogenous inducer of ferroptosis, and increasing YTHDC2 levels is another ferroptosis-based treatment strategy for LUAD [22].

According to these findings, m^6^A molecules and ferroptosis genes are associated with the prognosis of LUAD. There is a potential link between m^6^A and ferroptosis in LUAD. Therefore, we hypothesized that the existence of m^6^A-related ferroptosis genes (MRFGs) is related to the overall survival of patients with LUAD. To test this hypothesis, we identified six MRFGs as potential predictive biomarkers and constructed prognostic models based on these six MRFGs using bioinformatics methods.

## Materials and methods

### Data source and analysis

**Fig. 1:**
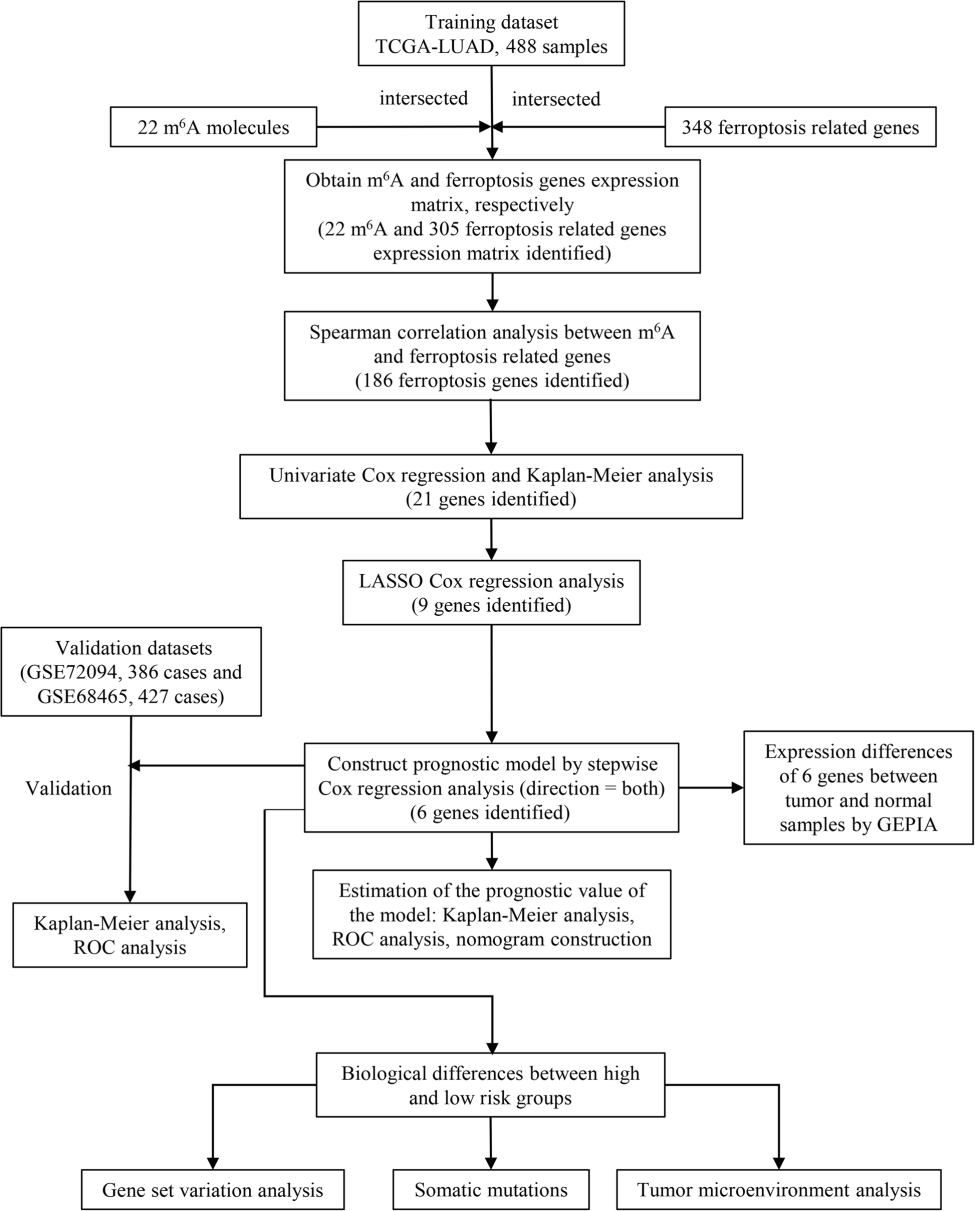
Flowchart of the study methodology

The RNA-seq fragments per kilobase million (FPKM) information on LUAD and related clinical data were obtained from University of California Santa Cruz (UCSC) Xena (http://xena.ucsc.edu/). Preliminary processing was performed according to the following criteria: [1] genes with zero expression in more than 30 samples were excluded; [2] samples that contained expression profiles but no clinical information or prognostic data were excluded; and [3] samples with a follow-up of < 30 days were removed. We screened 488 patients with LUAD from the UCSC Xena database as the training cohort. Mutation data were downloaded from the Genomic Data Commons Data Portal (https://portal.gdc.cancer.gov/). Two datasets (GSE72094 and GSE68465) were also downloaded as validation cohorts from the Gene Expression Omnibus (GEO) (https://www.ncbi.nlm.nih.gov/geo/) database. The final GSE72094 (n = 386) and GSE68465 (n = 427) datasets were used as validation cohorts (Supplementary Table 1). The clinical baseline characteristics of the three datasets are summarized in Table 1. Ferroptosis genes were downloaded from the FerrDb database (http://www.datjar.com:40013/bt2104/) and 348 ferroptosis-related genes were screened (Supplementary Table 2). The study flowchart is shown in Fig. 1.

**Table 1:** Clinical baseline characteristics of the three cohorts

Clinical characteristics	LUAD (n = 488)	GSE72094 (n = 386)	GSE68465 (n = 427)
Age			
<=65	238(48.8%)	115(29.8%)	223(52.2%)
> 65	250(51.2%)	271(70.2%)	204(47.8%)
Gender			
Male	228(46.7%)	168(43.5%)	216(50.6%)
Female	260(53.3%)	218(56.5%)	211(49.4%)
Tumor stage			
Stage I	265(54.3%)	246(63.7%)	NA
Stage II	114(23.4%)	65(16.8%)	NA
Stage III	77(15.8%)	56(14.5%)	NA
Stage IV	25(5.1%)	14(3.6%)	NA
Unknown	7(1.4%)	5(1.3%)	NA
T stage			
T1	164(33.6%)	NA	146(34.2%)
T2	259(53.1%)	NA	241(56.4%)
T3	44(9.0%)	NA	27(6.3%)
T4	18(3.7%)	NA	11(2.6%)
Unknown	3(0.6%)	NA	2(0.5%)
N stage			
N0	320(65.6%)	NA	290(67.9%)
N1	90(18.4%)	NA	82(19.2%)
N2	66(13.5%)	NA	52(12.2%)
N3	2(0.4%)	NA	0(0%)
Unknown	10(2.0%)	NA	3(0.7%)
Tobacco smoking history			
Ever	336(68.9%)	291(75.4%)	285(66.7%)
Never	0(0%)	30(7.8%)	49(11.5%)
Unknown	152(31.1%)	65(16.8%)	93(21.8%)
Race			
White	384(78.7%)	365(94.6%)	282(66.0%)
Non-white	60(12.3%)	18(4.7%)	18(4.2%)
Unknown	44(9.0%)	3(0.7%)	127(29.7%)

### Selection of m^6^A molecules and MRFGs

We extracted 22 m^6^A molecules and 305 ferroptosis gene expression profiles from the LUAD gene expression profiles. The following 22 molecules were defined as m^6^A molecules: writers (METTL3, METTL14, METTL16, WTAP, VIRMA, ZC3H13, RBM15, and RBM15B), readers (YTHDC1, YTHDC2, YTHDF1, YTHDF2, YTHDF3, HNRNPC, FMR1, LRPPRC, HNRNPA2B1, IGFBP2, IGFBP3, and RBMX), and erasers (FTO and ALKBH5). The correlation between the expression levels of m^6^A molecules and ferroptosis-related genes was analyzed using Spearman’s correlation analysis. We identified MRFGs based on the correlation between the expression of ferroptosis genes and the 22 m^6^A molecules with expression levels > 0.3 (|Spearman R | > 0.3 and *P* < 0.001).

### Construction and validation of the prognostic gene signature

We used the UCSC-Xena dataset as the training cohort and the two GEO datasets as the validation cohort. In the training cohort, univariate Cox regression and Kaplan–Meier analyses were used to identify potential prognostic genes. These prognostic genes were further screened using Lasso regression analysis by R packages “glmnet” [23], and the penalty parameter lambda was adjusted by 10-fold cross-validation. Prognostic genes were identified based on the best lambda value. Finally, the genes obtained from the Lasso analysis were entered into a stepwise Cox regression analysis (direction = both) to screen hub prognostic genes and construct the optimal prognostic gene signature. The following risk score formula was obtained from the gene signature:

$$Risk\;score={\textstyle\sum_{i=1}^n}Expi\;\ast\;\beta i$$

where *n, Expi*, and *βi* indicate the number of hub genes, gene expression level, and stepwise Cox regression coefficient, respectively. In the training cohort, patients were divided into high- and low-risk groups based on the median risk score, and the difference in prognosis between the two groups was assessed using the Kaplan–Meier analysis. We used univariate and multivariate Cox regression analyses between the risk score and clinical characteristics (gender, age, and stage) to assess whether the risk score was an independent prognostic factor. We conducted a time-receiver operating characteristic (time-ROC) analysis and constructed a nomogram to further assess the prognostic predictive power of the risk score. In the validation cohorts, the same formula and statistical methods (Kaplan–Meier analysis and time-ROC) were used to validate the prognostic power of the gene signature.

### Gene set variation analysis

Gene set variation analysis (GSVA) is used to estimate changes in pathway activity in a sample population in an unsupervised manner, allowing for a better detection of subtle changes in pathway activity [24]. To explore differences in underlying molecular signaling mechanisms (kyoto encyclopedia of genes and genomes [25], gene ontology) between the high- and low-risk groups, data from c2.cp.kegg.v7.4.symbols and c5.go.v7.4.symbols were downloaded from the molecular signatures database (MSigDB) (http://www.gsea-msigdb.org/gsea/msigdb/index.jsp). GSVA was used to evaluate the differences in biological functions between the two risk groups. |Log2(FC)| > 0.20 and *P* < 0.001 were set to indicate pathway activation.

### Assessing somatic mutations and tumor microenvironment characteristics

To explore the differences in somatic mutations between the high- and low-risk groups, we used the R package “maftool” [26] to calculate somatic mutations between the two groups. Using the R package “estimate” [27], we implemented the ESTIMATE algorithm to obtain scores for tumor purity, level of stromal cell presence, and level of immune cell infiltration in tumor tissue based on expression data. The ESTIMATE method was used to evaluate the immune/stromal/estimate scores for each lung cancer sample. The differences in the immune/stromal/estimate scores were then compared between the high- and low-risk groups. The CIBERSORT algorithm is a deconvolution method that characterizes the cell composition of complex tissues using gene expression profiles [28]. A machine learning algorithm (linear support vector regression) is used to deconvolute the mixture of gene expression. We calculated the abundance of the 22 immune cell infiltrates for each lung cancer sample using the CIBERSORT algorithm and compared the differences in the levels of 22 tumor immune infiltrate cells (TIICs) between the high- and low-risk groups.

### Statistical analysis

The R (v3.6.3) software was used for data processing and statistical analyses. Quantitative data were compared between two groups using the Wilcoxon test. Quantitative data among the three groups were compared using the Kruskal–Wallis test. Qualitative data were analyzed using the chi-square test or Fisher’s exact test. Spearman’s correlation analysis was used to analyze the correlation between m^6^A molecules and ferroptosis genes. The R package “survival” [29] was used for the Kaplan–Meier analysis and log-rank test. Stepwise Cox regression analyses and prognostic gene signature constructions were applied using the R package “survival”. Univariate and multivariate Cox regression analyses were conducted using the R package “survival”. ROC curves and area under the curve (AUC) calculations were performed using the R package “timeROC” [30]. A nomogram was constructed using the R package “rms” [31]. Calibration curves were analyzed using the bootstrap method to assess the predictive performance of the nomogram. *P* < 0.05 was considered statistically significant.

## Results

### Identification of MRFGs signature

**Fig. 2:**
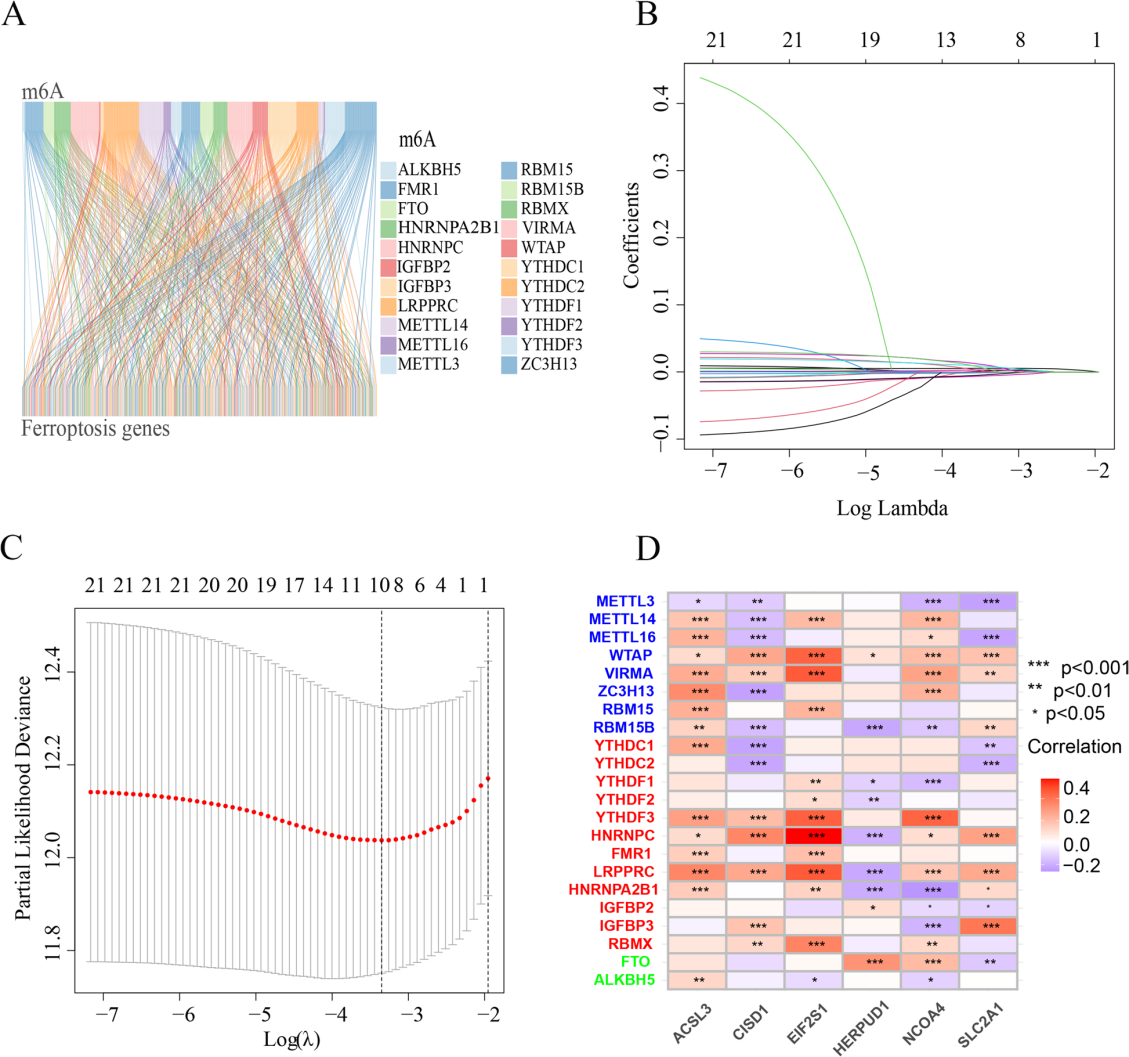
Gene signature obtained based on the m^6^A-related ferroptosis gene. **A** Sankey diagram showing the expression network relationship between the 22 m^6^A molecules and 186 m^6^A-related ferroptosis genes. **B** Lasso coefficient profiles of the 21 m^6^A-related ferroptosis prognostic genes. **C** Ten-fold cross-validation for the optimal parameter selection in the Lasso regression. **D** Heatmap plots of the correlations of the 22 m^6^A molecules with the six prognostic m^6^A-related ferroptosis genes (*P < 0.05, **P < 0.01, ***P < 0.001)

**Fig. 3:**
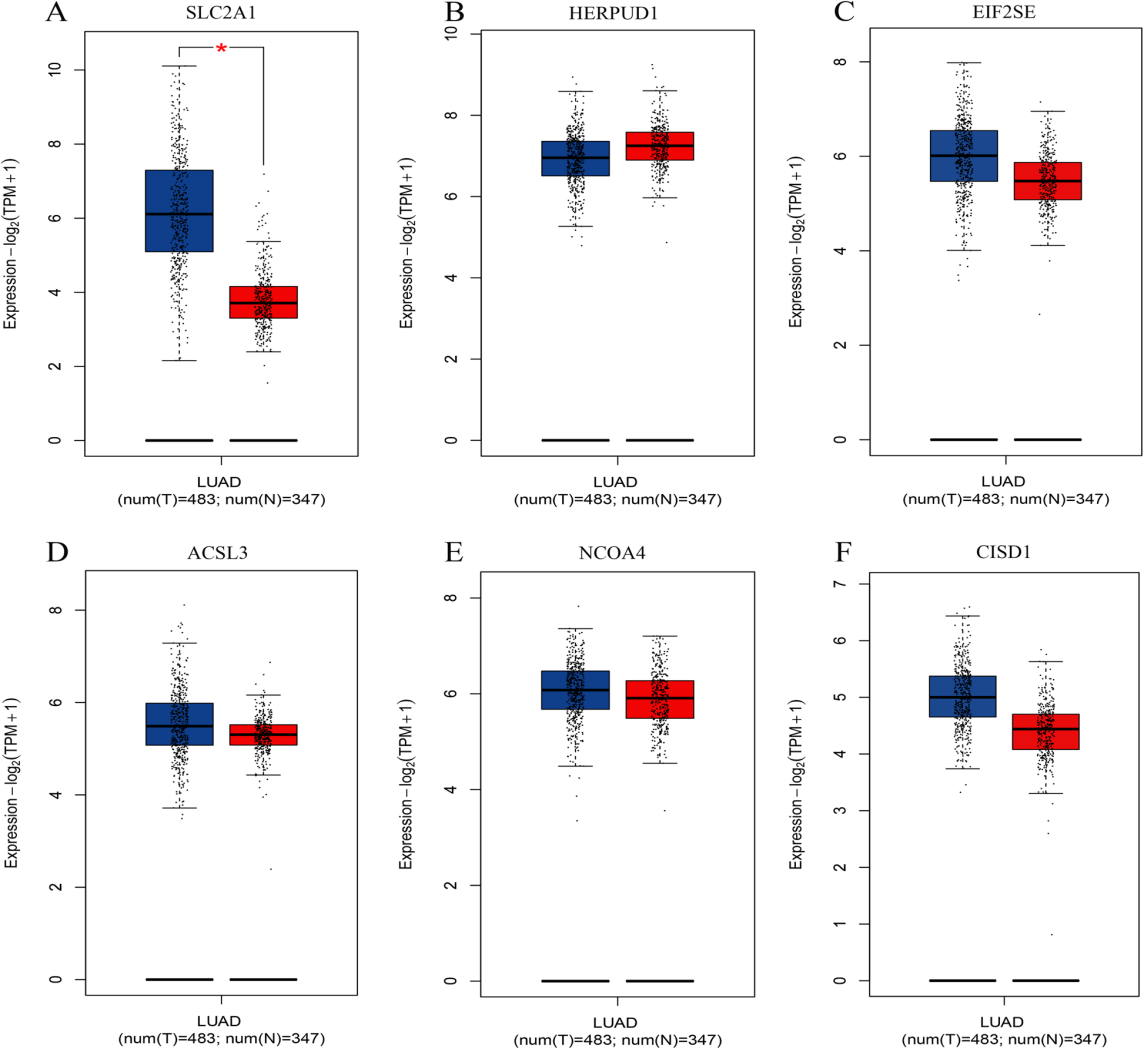
Expression levels of six genes in tumor and normal tissues evaluated using the GEPIA database. **A** SLC2A1, (**B**) HERPUD1, (**C**) EIF2S1, (**D**) ACSL3, (**E**) NCOA4, and (**F**) CISD1. Green represents the tumor samples and red represents the normal samples (*P < 0.05)

We obtained 186 MRFGs and visualized their co-expression relationships using the Sankey diagram (Fig. 2A). We identified 21 potential m^6^A-related ferroptosis prognosis genes using univariate Cox regression and Kaplan–Meier analyses (Supplementary Table 3). These 21 genes were entered into the Lasso analysis and nine genes were acquired (lambda.min = 0.022) and entered into the stepwise Cox regression analysis to identify six hub prognostic genes (SLC2A1, HERPUD1, EIF2S1, ACSL3, NCOA4, and CISD1) and construct a prognostic model (Fig. 2B, C). Correlations between the 22 m^6^A molecules and six hub prognostic genes were visualized using a correlation heatmap (Fig. 2D). We used the GEPIA database (http://gepia.cancer-pku.cn/index.html) to compare the differences in expression of the six genes between the patients with LUAD and normal samples. We found that SLC2A1 was highly expressed in tumor samples (*P* < 0.05, Fig. 3A), and the expression of the other five genes was not significantly different between tumor and normal tissues (*P* > 0.05, Fig. 3B-F).

### Estimation of the prognostic value of the model in the training cohort

**Fig. 4:**
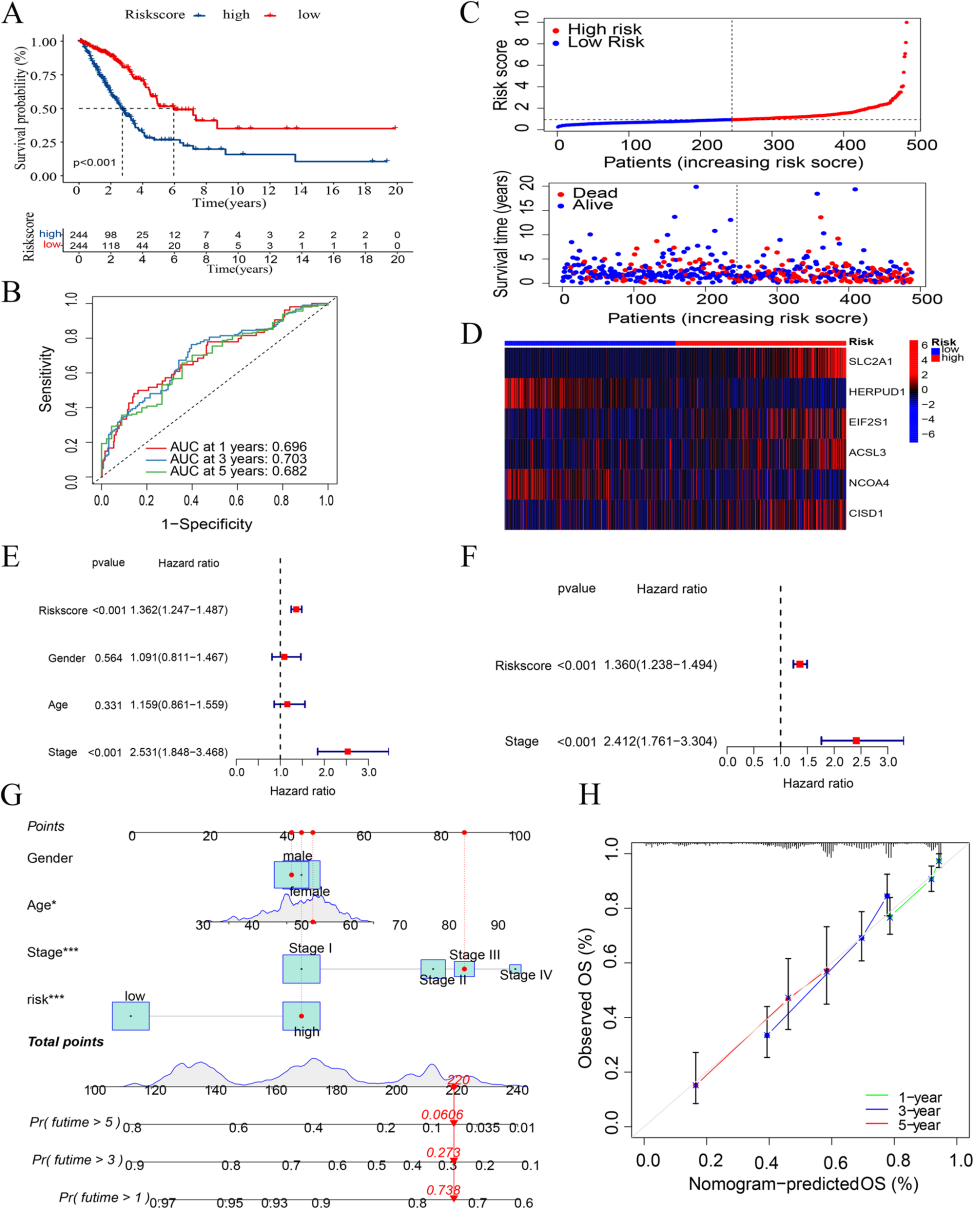
Prognostic value of the risk model signature. **A** Kaplan–Meier analysis of the prognosis in the low- and high-risk groups. **B** Prognostic ability of the risk score according to the time-ROC curve analysis. **C** Distribution of risk classes and survival time between the two groups. **D** Heatmap of the expression levels of the six genes. **E** Univariate Cox regression analysis of the risk score. **F** Multivariate Cox regression analysis of the risk score. **G** Nomogram predicting 1-, 3-, and 5-year survival outcomes. **H** Calibration plot of the nomogram to predict 1-, 3-, and 5-year survival

Patients were divided into high- and low-risk groups according to the median risk score, and worse clinical outcomes were seen in the high-risk group (*P* < 0.001, Fig. 4A, Supplementary Tables 4-S1). Patients were also divided into high- and low groups according to the median expression of genes, and the relationship between each gene and the prognosis of the patients was evaluated. Four genes with high expression were associated with poor prognosis (SLC2A1, *P* < 0.001; EIF2S1, *P* < 0.05; ACSL3, *P* < 0.01; CISD1, *P* < 0.01; Supplementary Fig. 1A-D), while two genes with high expression were associated with better prognosis (HERPUD1, *P* < 0.001; NCOA4, *P* < 0.01; Supplementary Fig. 1E, F). A time-ROC curve analysis was conducted to predict patients’ prognosis at 1, 3, and 5 years (AUC = 0.696, 0.703, and 0.682, respectively; Fig. 4B, Supplementary Tables 4-S1). The distribution of the risk classes and survival time between the high- and low-risk groups is shown in Fig. 4C (Supplementary Tables 4-S1). A heatmap was used to visualize the expression levels of the six genes for each patient (Fig. 4D, Supplementary Tables 4-S1). Univariate and multivariate Cox regression analyses showed that the risk score was an independent risk factor for prognosis (univariate: HR = 1.362, 95% CI: 1.247–1.487, *P* < 0.001 and multivariate: HR = 1.360, 95% CI: 1.238–1.494, *P* < 0.001; Fig. 4E-, F, Supplementary Tables 4-S2). According to the prognostic analysis in the two groups stratified by gender (female and male), age (≤ 65 and > 65 years), and stage (stages I–II and III–IV), the high-risk group had worse outcomes (Supplementary Fig. 2A-F). To facilitate use of the risk score, a nomogram was constructed with the risk score and clinical factors (gender, age, and stage) (Fig. 4G, Supplementary Tables 4-S3). Calibration plots for overall survival at 1, 3, and 5 years were used to visualize nomogram performance (Fig. 4H, Supplementary Tables 4-S3).

### Validation model stability on the GEO dataset

**Fig. 5:**
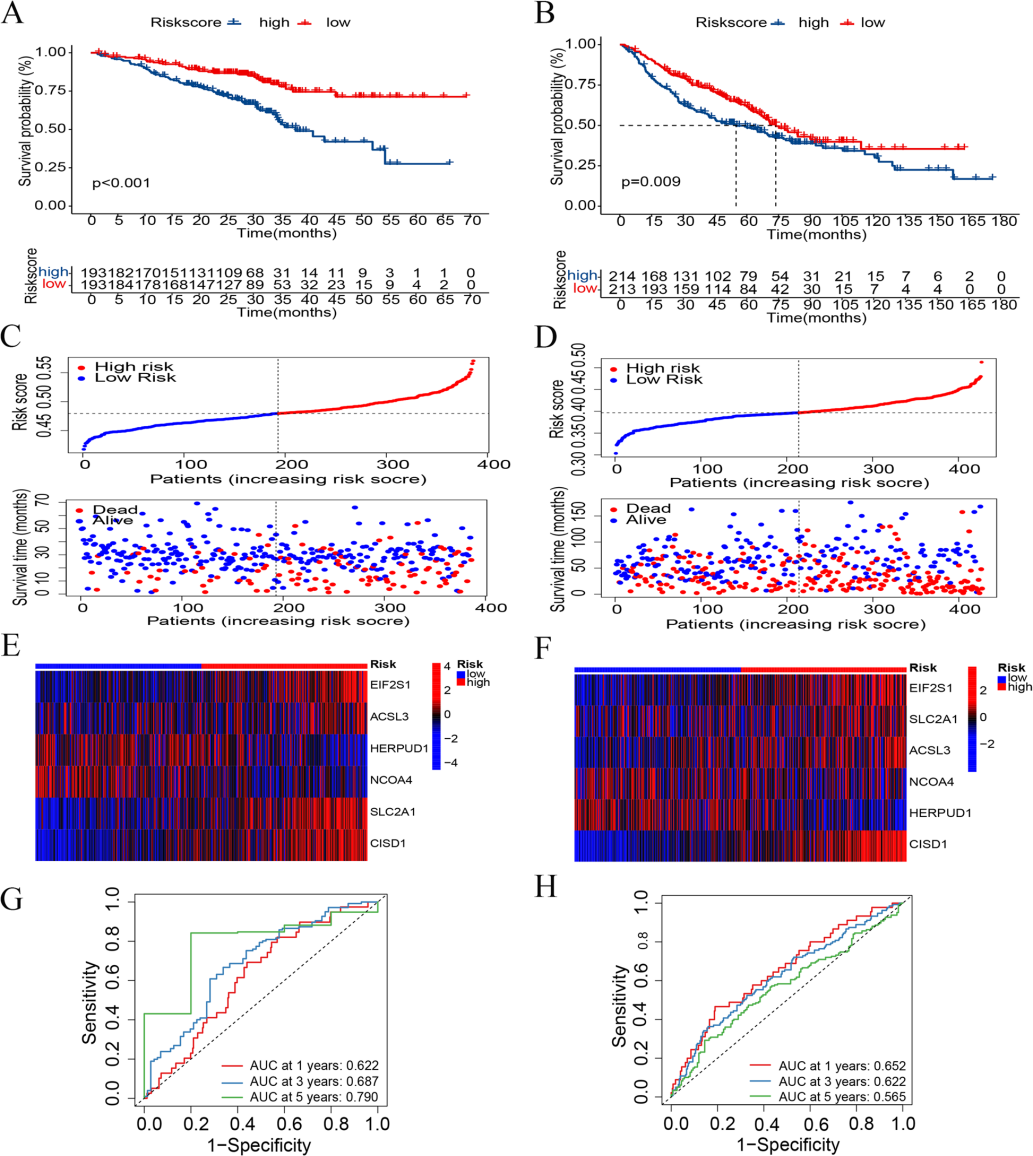
Validation model stability on the GEO datasets (GSE72094 and GSE68465). **A** Kaplan–Meier analysis between the high- and low-risk groups in the GSE72094 cohort. **B** Kaplan–Meier analysis between the high- and low-risk groups in the GSE68465 cohort. **C** Distribution of risk classes and survival time between the two groups in the GSE72094 cohort. **D** Distribution of risk classes and survival time between the two groups in the GSE68465 cohort. **E** Heatmap of the expression levels of the six genes in the GSE72094 cohort. **F** Heatmap of the expression levels of the six genes in the GSE68465 cohort. **G** Time-ROC curve analysis of the risk score in the GSE72094 cohort. **H** Time-ROC curve analysis of the risk score in the GSE68465 cohort

To validate the prognostic stability of the gene signature, two GEO datasets (GSE72094 and GSE68465) were used as the validation cohorts (Supplementary Table 5). The same formula that was used to calculate the risk score for the training cohort was applied to the GEO cohorts. According to the median risk score, patients were divided into high- and low-risk groups, and survival analyses showed that patients in the high-risk group had worse prognoses (GSE72094: *P* < 0.001 and GSE68465: *P* = 0.009; Fig. 5A, B). The distribution of risk classes and survival times between the two groups are shown in Fig. 5C, D. A heatmap was used to visualize the expression levels of the six genes for each patient (Fig. 5E, F). A time-ROC curve analysis was used to predict patients’ prognosis at 1, 3, and 5 years (GSE72094: AUC = 0.622, 0.687, and 0.790, respectively, and GSE68465: AUC = 0.652, 0.622, and 0.565, respectively; Fig. 5G, H). Principal component analysis (PCA) and t-distributed stochastic neighbor embedding (t-SNE) further confirmed that the risk score could be used to significantly distinguish between patients (Supplementary Fig. 3A-D). In general, the verification results showed that the gene signature had good stability.

### GSVA

**Fig. 6:**
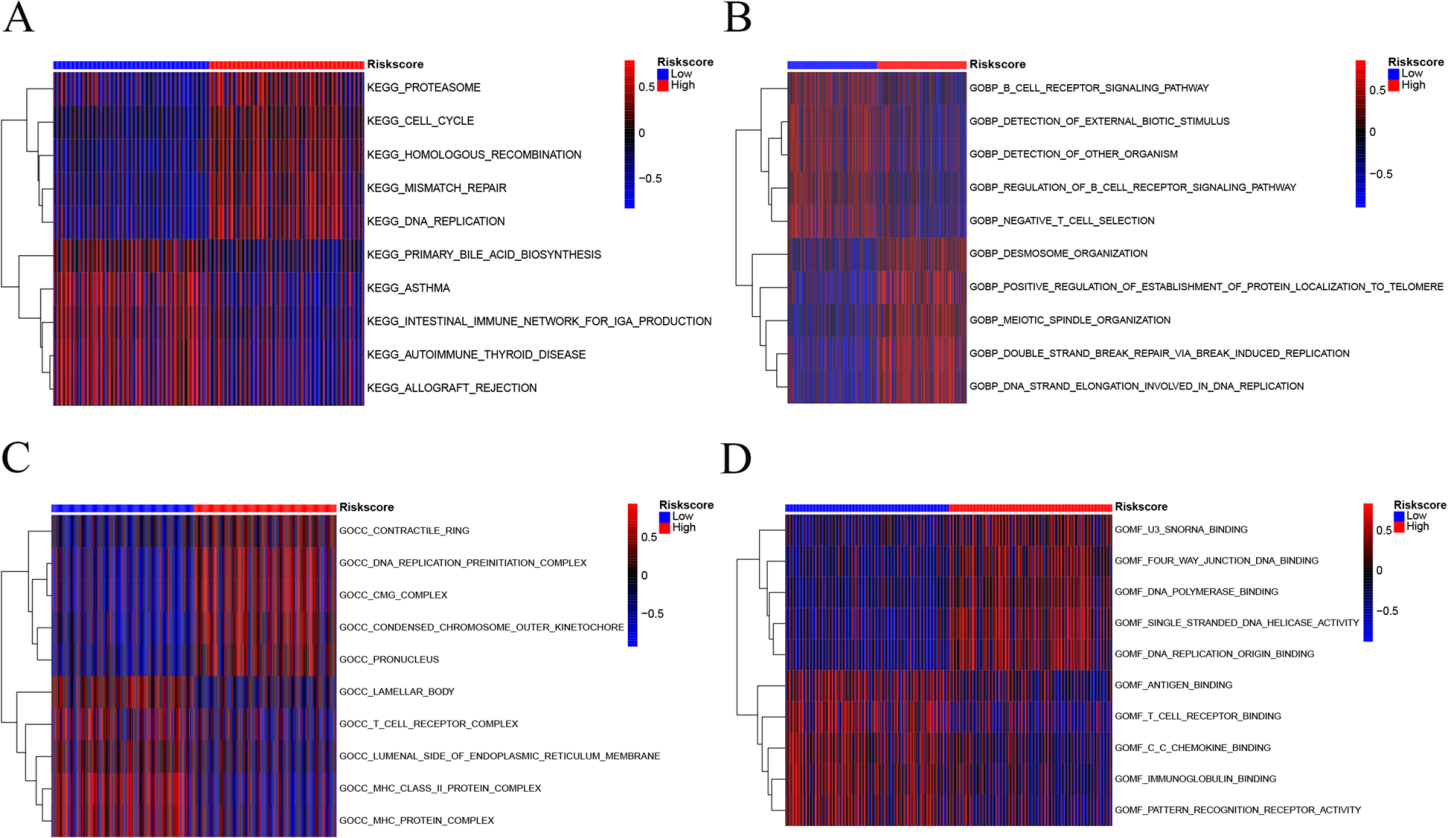
Enriched pathway differences between the high- and low-risk groups by GSVA. **A** Enriched pathway differences of KEGG between the two groups in c2.cp.kegg.v7.4.symbols. **B** Enriched pathway differences of GO-BP between the two groups in c5.go.v7.4.symbols. **C** Enriched pathway differences of GO-CC between the two groups in c5.go.v7.4.symbols. **D** Enriched pathway differences of GO-MF between the two groups in c5.go.v7.4.symbols

A GSVA was conducted to analyze the enriched pathways in the high- and low-risk groups to further explore the differences in participating gene ontology (GO) and Kyoto Encyclopedia of Genes and Genomes (KEGG) pathways between the two groups. In c2.cp.kegg.v7.4.symbols, we obtained the five most significantly correlated differential pathways based on log_2_(FC) values (Fig. 6A, Supplementary Tables 6-S1). We found that the high-risk group was mainly correlated with the upregulation of cell-cycle pathways (e.g., DNA replication, homologous recombination, mismatch repair, proteasome, cell cycle). In contrast, the low-risk group showed more upregulation of certain immune diseases (e.g., primary bile acid biosynthesis, asthma, the intestinal immune network for IgA production, autoimmune thyroid disease, and allograft rejection). In addition, the GO gene-set variation analysis in c5.go.v7.4.symbols also revealed that the patients in the high-risk group were had more upregulation of DNA replication, while the patients in the low-risk group had more upregulation of immune regulation (Fig. 6B-D, Supplementary Tables 6-S2).

### Somatic mutations analysis

**Fig. 7:**
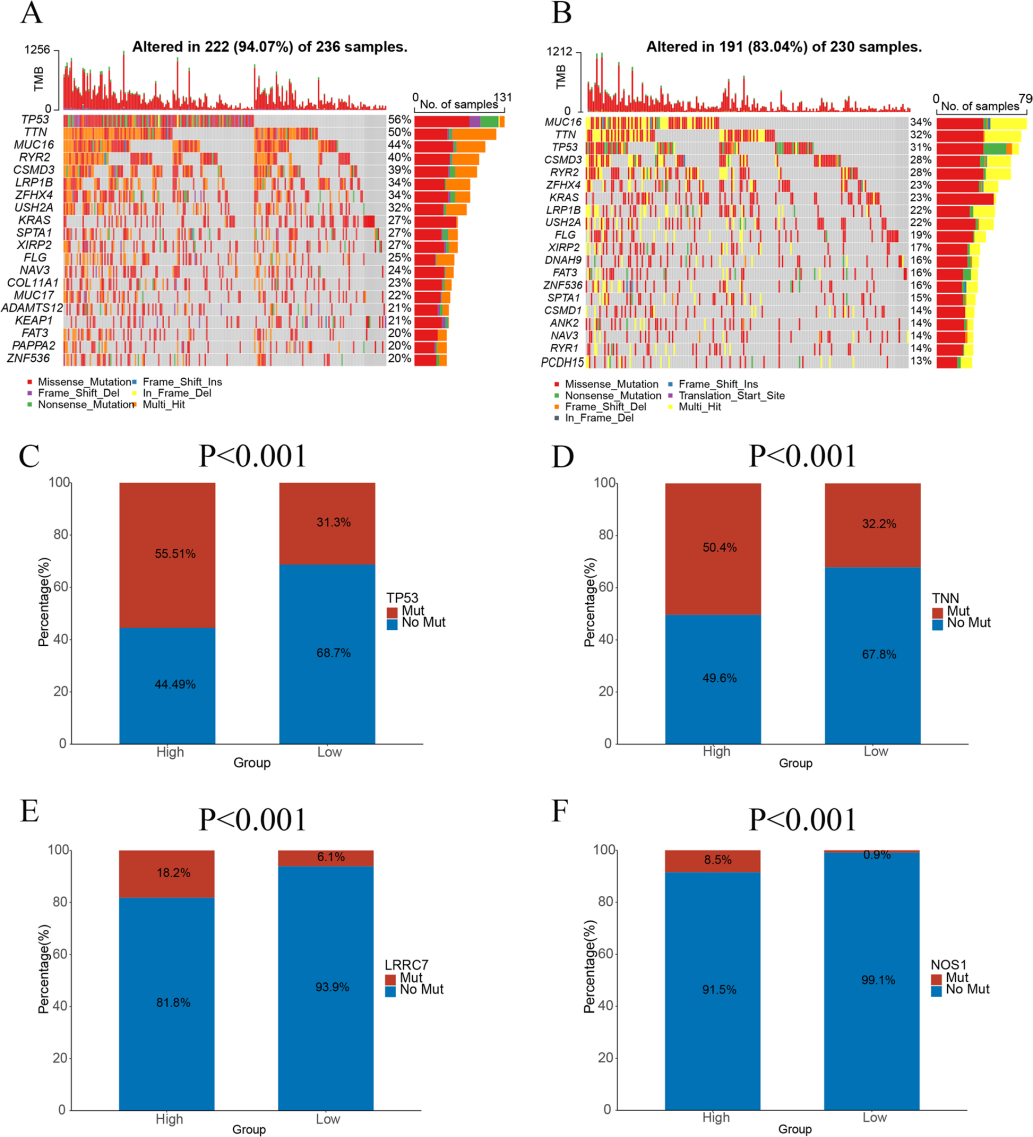
Somatic mutation analyses in the high- and low-risk groups. **A** Waterfall plot somatic mutation in the high-risk group. **B** Waterfall plot somatic mutation in the low-risk group. **C** Somatic mutation differences of the TP53 gene between the two groups. **D** Somatic mutation differences of the TNN gene between the two groups. **E** Somatic mutation differences of the LRRC7 gene between the two groups. **F** Somatic mutation differences of the NOS1 gene between the two groups. Red represents mutation and blue represents no mutation

To explore differences in somatic mutations between the high- and low-risk groups, we used waterfall plots to visualize the top 20 genes with the highest mutation frequencies in the two groups (Fig. 7A, B, Supplementary Tables 7-S1, 2). We further compared the mutational differences of all genes between the two groups, and the results showed that TP53, TNN, LRRC7, and NOS1 had more mutations in the high-risk group than in the low-risk group. The TP53 gene had the highest mutation rate in the high-risk group (Fig. 7C-F, Supplementary Tables 7-S3).

### Analysis of immune infiltration in the tumor microenvironment

**Fig. 8:**
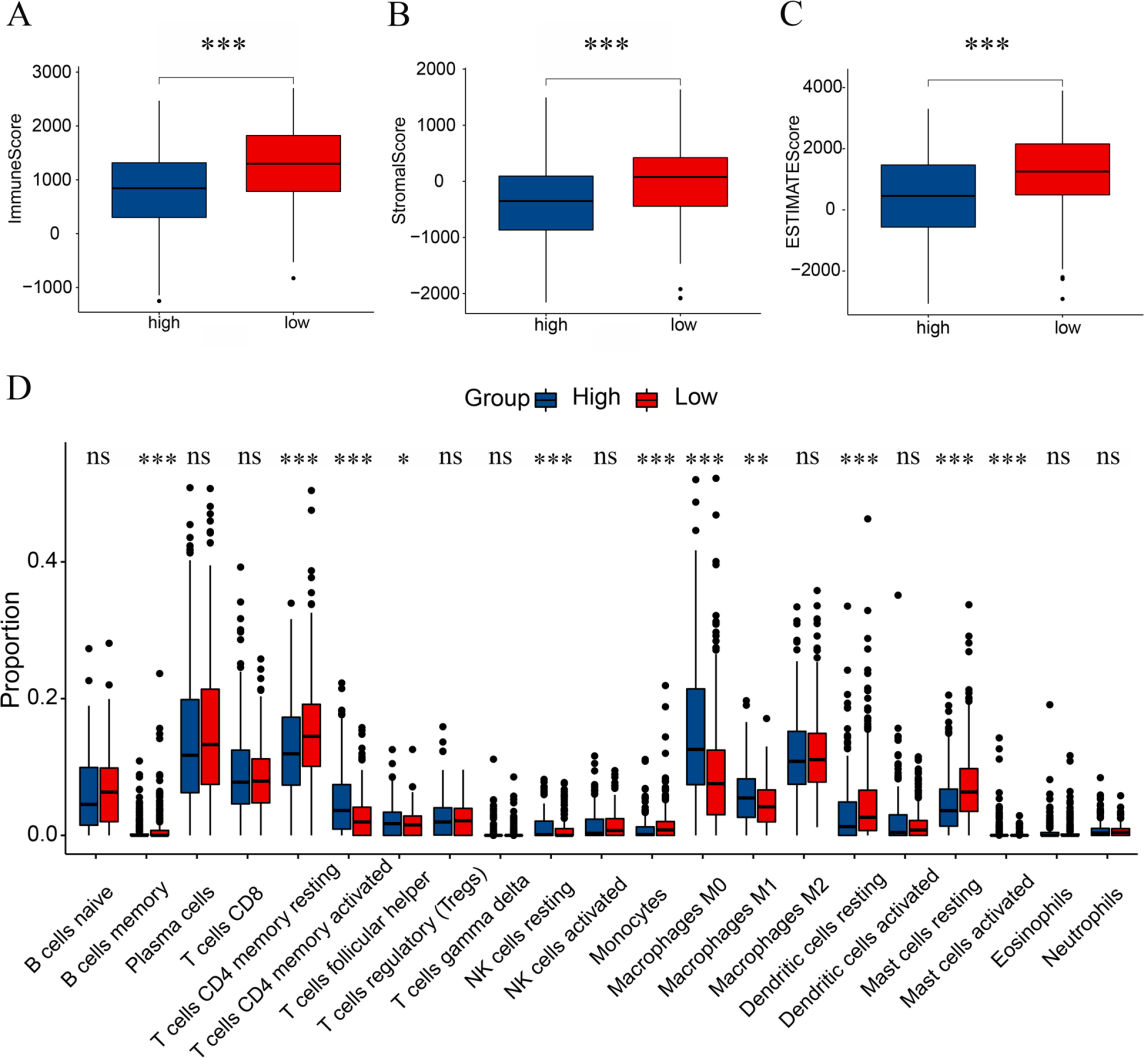
Analysis of tumor immune infiltration cells in the tumor microenvironment. **A** Differences in stromal scores among the high- and low-risk groups. **B** Differences in immune scores among the high- and low-risk groups. **C** Differences in ESTIMATE scores among the high- and low-risk groups. **D** Abundance of the 21 tumor immune infiltration cells in the high- and low-risk groups (ns, no significance, *P < 0.05, **P < 0.01, ***P < 0.001)

We aimed to explore differences in immune infiltration in the tumor microenvironment (TME) between the high- and low-risk groups. We used the ESTIMATE algorithm to calculate the distribution between the stromal/immune/estimate scores for patients in the high- and low-risk groups. Compared with the high-risk group, the low-risk group exhibited higher immune/stromal/estimate scores (Fig. 8A-C, Supplementary Tables 8-S1). The CIBERSORT algorithm showed that the high-risk group had higher levels of activated CD4 memory T cells, follicular helper T cells, resting NK cells, and M1 and M0 macrophages, while the low-risk group had higher levels of memory B cells, resting CD4 memory T cells, monocytes, resting dendritic cells, and resting mast cells (Fig. 8D, Supplementary Tables 8-S2).

## Discussion

LUAD is a highly heterogeneous malignancy [32] with a low 5-year survival rate [33]. Identifying target molecules and building a predictive signature of stability is conducive to early intervention and can prolong the survival time. This study was inspired by the latest research on the potential association between m^6^A and ferroptosis genes. For our study, we built an m^6^A-related ferroptosis six-gene signature to predict LUAD prognosis through joint TCGA and GEO database mining. The six-gene signature showed good predictive value for LUAD in the validation group. In contrast to previous studies that have identified prognostic genetic signatures in LUAD, we are the first to use m^6^A-related ferroptosis genes. The present study therefore provides additional directions for LUAD research.

We further analyzed the biological functions of these six genes. SLC2A1 encodes a glucose transporter that controls glucose uptake, which can stimulate fatty acid synthesis and ultimately lead to cellular lipid peroxidation-dependent ferroptosis [34]. Studies have found that the m^6^A reader YTHDC1 is involved in suppressing the expression of SLC2A1 [35]. Correlation analysis has shown that YTHDC1 is negatively correlated with SLC2A1 (r = -0.15, *P* < 0.01). SLC2A1 overexpression can promote the growth and proliferation of various tumor cells [36–39] and is associated with poor prognosis in lung cancer [36]. In this study, SLC2A1 overexpression was associated with poorer clinical prognoses in patients with LUAD (*P* < 0.001). HERPUD1 is an endoplasmic reticulum protein processing-encoding gene. Studies have reported that HERPUD1 overexpression can promote apoptosis of various cancer cells (e.g., gastric, prostate, and endometrial cancer) induced by endoplasmic reticulum stress [40–42]. The results of this study showed a better prognosis for patients with lung cancer that have high HERPUD1 expression. EIF2S1 (eIF2α) is a translation initiation factor that causes global arrest in protein synthesis via phosphorylation in eukaryotic cells [43, 44]. Avitan-Hersh et al. confirmed that eIF2α is involved in the occurrence and treatment resistance of melanoma [45]. Bai et al. demonstrated that activation of the eIF2α/ATF4 pathway is involved in radioresistance in triple-negative breast cancer [46]. Additionally, Jeon et al. verified that TIPRL can prolong survival in patients with lung cancer by inducing autophagy through the eIF2α-ATF4 axis [47]. Increased eIF2α phosphorylation is associated with poor prognosis in patients with LUAD [48]. Our results indicate a worse prognosis for patients with LUAD who have high expression of EIF2S1. ACSL3 plays an important role in fatty acid metabolism [49] and can inhibit ferroptosis to protect the cells [50]. ACSL3 overexpression results in worse clinical prognosis in high-grade NSCLC [51]. NCOA4 is a selective cargo receptor for the autophagic degradation of ferritin that weakens ferroptosis [52]. Studies have reported that high expression of NCOA4 is associated with prolonged overall tumor survival [53, 54]. The results of this study also showed that highly expressed NCOA3 is associated with better clinical prognosis, though the mechanism is still unclear. CISD1 mediates mitochondrial lipid peroxidation to inhibit ferroptosis [55], which plays an important role in promoting cancer cell proliferation and supporting tumor development and metastasis [56]. However, the biological functions of CISD1 in LUAD remain unclear.

GSVA and immune infiltration analysis showed higher immune activity in the low-risk group than in the high-risk group. Studies have reported that the mechanism of immune checkpoint inhibitors involves unblocking certain inhibitory pathways, thereby enhancing the immune system to produce antitumor activity [57]. Somatic mutation analysis showed the TP53 gene had the most significant mutation rate in the high-risk group compared to the low-risk group. TP53 mutations in LUAD have been associated with significantly higher levels of antitumor immune features than TP53 wild-type cancers [58].

The CIBERSORT algorithm was used to analyze differences in TIICs between the high- and low-risk groups. Both groups had higher levels of resting CD4 memory cells and M0 macrophages relative to other infiltrating cells. Compared with the high-risk group, the low-risk group had higher levels of resting CD4 memory T cells and lower levels of M0 macrophages. Quiescent CD4 memory T cells have been found to differentiate and confer multiple functions, such as assisting CD8 + T cells with performing antitumor functions [59]. An increased number of M0 macrophages is associated with poor prognosis in LUAD at an early clinical stage [60]. These results suggest that the tumor immune response mechanisms may differ between the two groups.

This study has some limitations. First, the clinical samples (three cohorts) used for prognostic feature construction and validation were sourced from public databases. This gene signature would be more reliable if tested in a prospective clinical trial cohort. Secondly, the biological mechanisms of action of m^6^A molecules associated with the six ferroptosis genes have not been elucidated, and further experimental evidence is needed to validate the association of m^6^A with these six core prognostic genes and ferroptosis’ regulatory function in LUAD.

## Conclusions

In conclusion, our study identified a robust m^6^A-related ferroptosis six-gene signature that predicts LUAD prognosis. Notably, we validated the reliability and applicability of the signature using two independent validation cohorts. Our findings provide useful biomarkers for LUAD prognostic prediction and insights for identifying new molecules or targets for LUAD therapy.

## Supplementary Information

**Additional file 1: Supplementary Table 1.** The raw data were applied to process the normalized GSE72094 expression profile.

**Additional file 2: Supplementary Table 2.** 348 ferroptosis genes were downloaded from the FerrDb database.

**Additional file 3: Supplementary Table 3.** Associations with overall survival and m6A related ferroptosis prognostic genes in LUAD patients using univariate Cox regression and Kaplan-Meier analyses.

**Additional file 4: Supplementary Table 4-S3.** The raw data used to generate Figure 4G-H.

**Additional file 5: Figure S1.** The relationship between six gene in the model and the prognosis of patients with LUAD.

**Additional file 6: Figure S2.** Survival analysis differences stratified by gender, age and stage in LUAD patients.

**Additional file 7: Supplementary Table 5.** The raw data used to generate Figure 5A,C,E,G.

**Additional file 8: Figure S3.** Differentiation of LUAD patients by PCA and t-SNE based on the risk model.

**Additional file 9: Supplementary Table 6-S2.** The raw data used to generate Figure 6B-D.

**Additional file 10: Supplementary Table 7-S3.** The raw data used to generate Figure 7C-F.

**Additional file 11: Supplementary Table 8-S1.** The raw data used to generate Figure 8A-C

## Data Availability

All data were downloaded from the following public databases: UCSC Xena (https://xenabrowser.net/datapages/?dataset=TCGA-LUAD.htseq_fpkm.tsv&host=https%3A%2F%2Fgdc.xenahubs.net&removeHub=https%3A%2F%2Fxena.treehouse.gi.ucsc.edu%3A443), Genomic Data Commons Data Portal (https://portal.gdc.cancer.gov/repository?facetTab=files&filters=%7B%22op%22%3A%22and%22%2C%22content%22%3A%5B%7B%22content%22%3A%7B%22field%22%3A%22cases.project.project_id%22%2C%22value%22%3A%5B%22TCGA-LUAD%22%5D%7D%2C%22op%22%3A%22in%22%7D%2C%7B%22content%22%3A%7B%22field%22%3A%22files.data_category%22%2C%22value%22%3A%5B%22Simple%20Nucleotide%20Variation%22%5D%7D%2C%22op%22%3A%22in%22%7D%2C%7B%22op%22%3A%22in%22%2C%22content%22%3A%7B%22field%22%3A%22files.data_format%22%2C%22value%22%3A%5B%22maf%22%5D%7D%7D%2C%7B%22op%22%3A%22in%22%2C%22content%22%3A%7B%22field%22%3A%22files.data_type%22%2C%22value%22%3A%5B%22Masked%20Somatic%20Mutation%22%5D%7D%7D%5D%7D&searchTableTab=cases) and Gene Expression Omnibus (https://ftp.ncbi.nlm.nih.gov/geo/series/GSE72nnn/GSE72094/matrix/), (https://ftp.ncbi.nlm.nih.gov/geo/series/GSE68nnn/GSE68465/matrix/).

## Abbreviations

m^6^A: N6-methyladenosine
LUAD: Lung adenocarcinoma
NSCLC: Non-small-cell lung cancer
MRFGs: m^6^A-related ferroptosis genes
GSVA: Gene set variation analysis
TIICs: Tumor immune infiltration cells
TME: Tumor microenvironment.
