# Supplementary material for: Identification of a m6A-related ferroptosis signature as a potential predictive biomarker for lung adenocarcinoma
Source: BMC Pulm Med. 2023 Apr 18;23:128. doi: 10.1186/s12890-023-02410-x (PMC10111681; doi:10.1186/s12890-023-02410-x)
Supplement: Supplementary file 3 — Additional file 3: Supplementary Table 3. Associations with overall survival and m6A related ferroptosis prognostic genes in LUAD patients using univariate Cox regression and Kaplan-Meier analyses. [file 12890_2023_2410_MOESM3_ESM.docx]

**SUPPORTING INFORMATION**

**Supplementary Table 3. Associations with overall survival and m^6^A related ferroptosis prognostic genes in LUAD patients using univariate Cox regression and Kaplan-Meier analyses.**

| **gene** | **Description** | **Kaplan-Meier analysis**  **(*P*-value)** | **Univariate Cox regression analysis** | | | |
| --- | --- | --- | --- | --- | --- | --- |
|  |  |  | **HR** | **HR_95L** | **HR_95H** | ***P*-value** |
| SLC2A1 | Solute carrier family 2 member 1 | 4.03E-05 | 1.008 | 1.006 | 1.011 | 1.104E-10 |
| ARNTL | Aryl hydrocarbon receptor nuclear translocator like | 0.014870328 | 0.862 | 0.758 | 0.980 | 0.023729281 |
| FLT3 | Fms related tyrosine kinase 3 | 0.003408397 | 0.471 | 0.260 | 0.853 | 0.012989641 |
| ARRDC3 | Arrestin domain containing 3 | 0.018020531 | 0.995 | 0.989 | 1.000 | 0.039770011 |
| YWHAE | Tyrosine 3 -monooxygenase/tryptophan  5-monooxygenase activation Protein Epsilon | 0.049843813 | 1.004 | 1.000 | 1.007 | 0.027051368 |
| HERPUD1 | Homocysteine-inducible ER protein with ubiquitin-like domain 1 | 0.000875427 | 0.981 | 0.970 | 0.992 | 0.000772833 |
| RRM2 | Ribonucleotide reductase regulatory subunit M2 | 0.000616267 | 1.023 | 1.012 | 1.035 | 8.07E-05 |
| PANX1 | Pannexin 1 | 0.007331173 | 1.038 | 1.009 | 1.069 | 0.010956983 |
| TRIB3 | Tribbles pseudokinase 3 | 0.019585739 | 1.012 | 1.001 | 1.023 | 0.029173657 |
| CYBB | Cytochrome b-245 beta chain | 0.020704408 | 0.992 | 0.986 | 0.999 | 0.026217666 |
| AURKA | Aurora kinase A | 0.016366047 | 1.022 | 1.007 | 1.037 | 0.004113128 |
| EIF2S1 | Eukaryotic translation initiation factor 2 subunit alpha | 0.036279794 | 1.043 | 1.016 | 1.071 | 0.001618536 |
| FANCD2 | FA complementation group D2 | 0.014559992 | 1.114 | 1.004 | 1.237 | 0.041880106 |
| IL33 | Interleukin 33 | 0.046656217 | 0.977 | 0.958 | 0.996 | 0.020036369 |
| ATP5MC3 | ATP synthase membrane subunit c locus 3 | 0.037491876 | 1.017 | 1.005 | 1.029 | 0.005969083 |
| TLR4 | Toll-like receptor 4 | 0.041213196 | 0.952 | 0.907 | 0.999 | 0.045770769 |
| ACSL3 | Acyl-CoA synthetase long-chain family member 3 | 0.006743979 | 1.021 | 1.008 | 1.035 | 0.001683939 |
| VDAC2 | Voltage-dependent anion channel 2 | 0.002685876 | 1.029 | 1.015 | 1.043 | 3.56E-05 |
| NCOA4 | Nuclear receptor coactivator 4 | 0.005309748 | 0.990 | 0.982 | 0.998 | 0.015524281 |
| CDCA3 | Solute carrier family 7 member 11 | 0.000215143 | 1.095 | 1.025 | 1.170 | 0.007089316 |
| CISD1 | CDGSH iron sulfur domain 1 | 0.009424265 | 1.043 | 1.003 | 1.085 | 0.034040026 |

KM: Kaplan-Meier; HR: hazard ratio; CI: confidence interval.
