# Supplementary material for: Identification of a m6A-related ferroptosis signature as a potential predictive biomarker for lung adenocarcinoma
Source: BMC Pulm Med. 2023 Apr 18;23:128. doi: 10.1186/s12890-023-02410-x (PMC10111681; doi:10.1186/s12890-023-02410-x)
Supplement: Supplementary file 5 — Additional file 5: Figure S1. The relationship between six gene in the model and the prognosis of patients with LUAD. [file 12890_2023_2410_MOESM5_ESM.docx]

**SUPPORTING INFORMATION**

**Figure S1. The relationship between six gene in the model and the prognosis of patients with LUAD.**


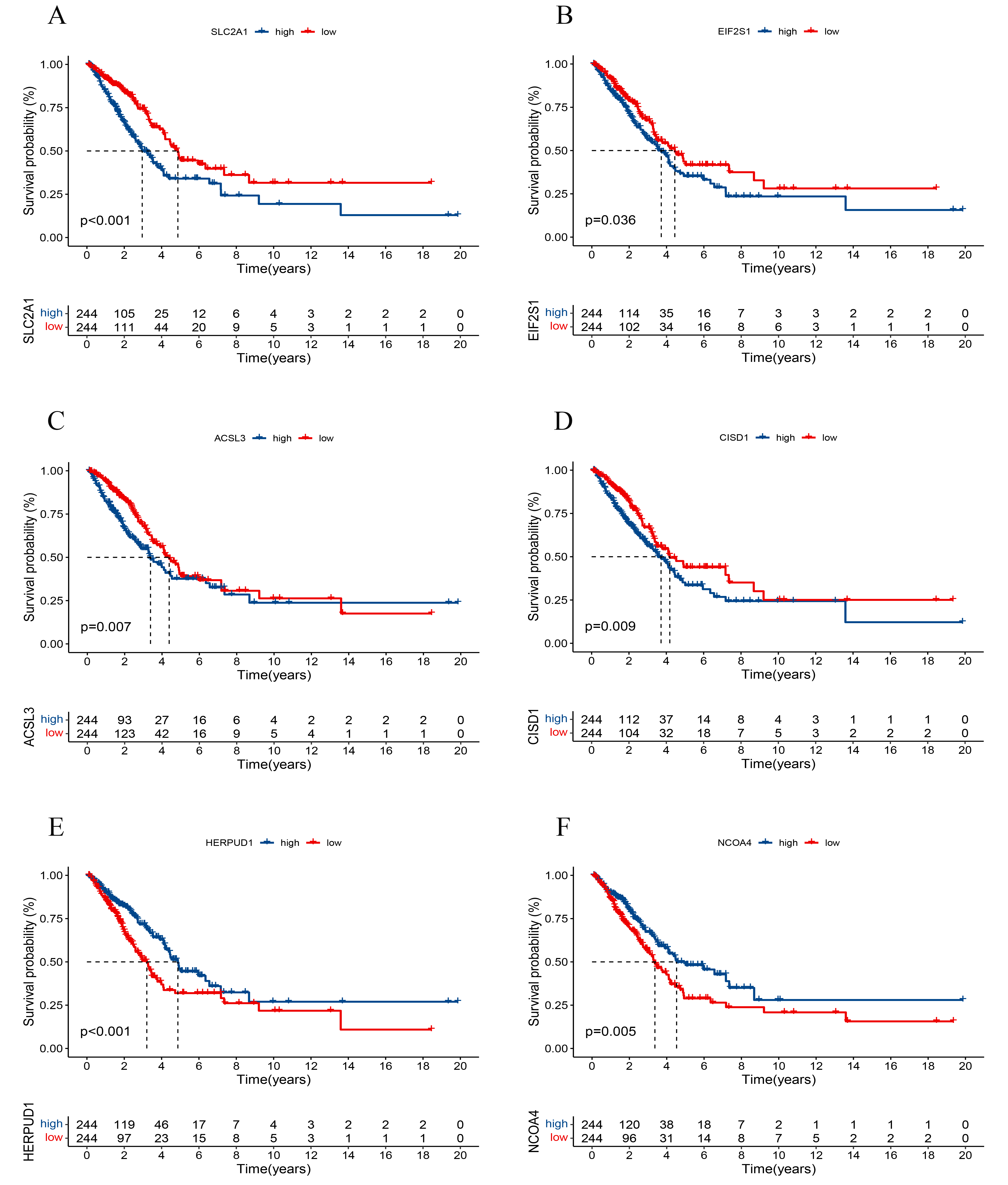


**Figure S1:** The relationship between six gene in the model and the prognosis in LUAD patients. Evaluating gene prognosis based on median gene expression. (A) SLC2A1. (B) EIF2S1. (C) ACSL3. (D) CISD1. (E) HERPUD1. (F) NCOA4.
