# Supplementary material for: Identification of a m6A-related ferroptosis signature as a potential predictive biomarker for lung adenocarcinoma
Source: BMC Pulm Med. 2023 Apr 18;23:128. doi: 10.1186/s12890-023-02410-x (PMC10111681; doi:10.1186/s12890-023-02410-x)
Supplement: Supplementary file 6 — Additional file 6: Figure S2. Survival analysis differences stratified by gender, age and stage in LUAD patients. [file 12890_2023_2410_MOESM6_ESM.docx]

**SUPPORTING INFORMATION**

**Figure S2. Survival analysis differences stratified by gender, age and stage in LUAD patients.**


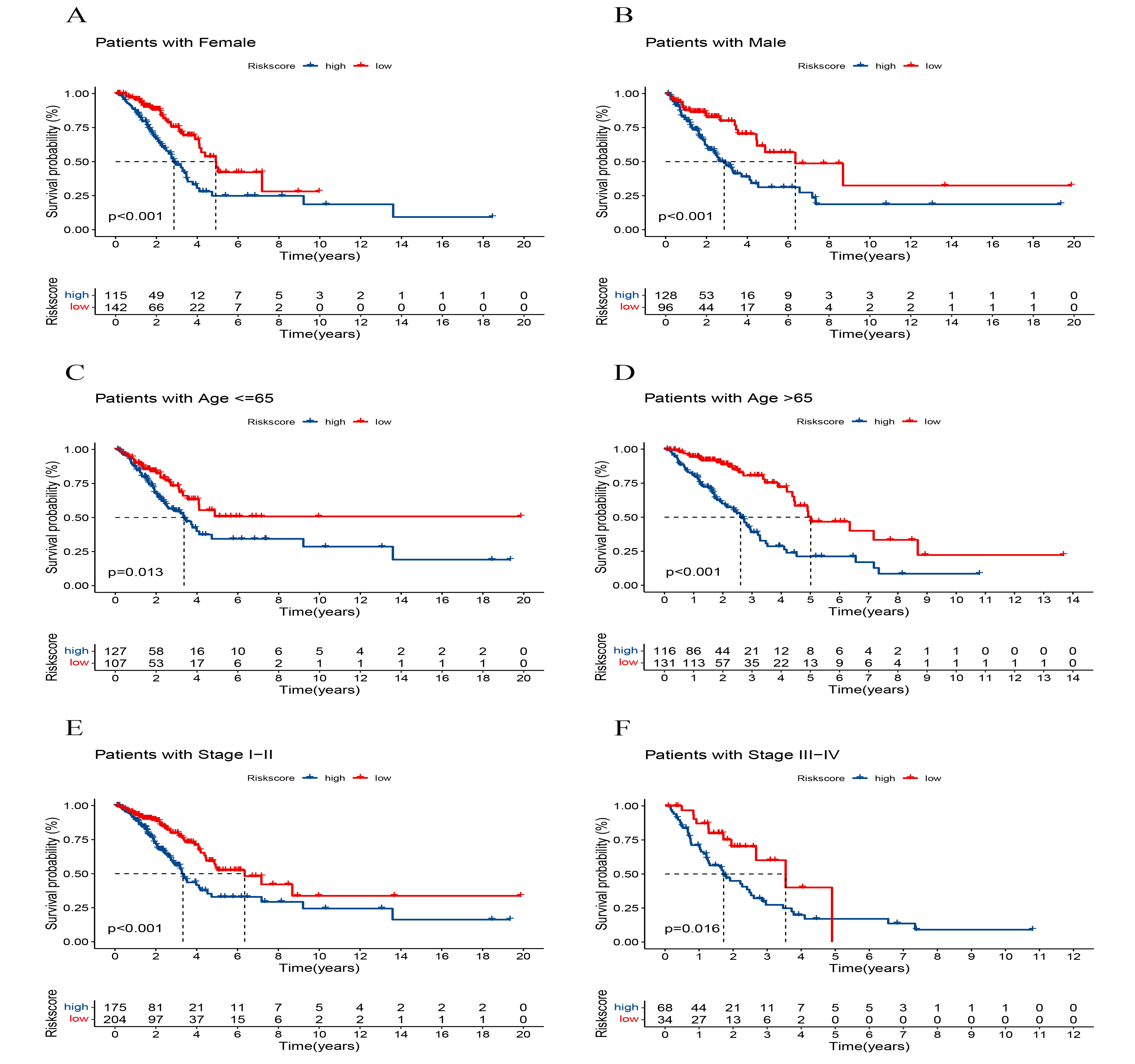


**Figure S2:** survival analysis differences stratified by gender (female and male), age (<=65 and >65), and stage (stage I-II and III-IV) between the low and high-risk groups. (A) Female. (B) Male. (C) Age <=65. (D) Age >65. (E) Stage I-II. (F) Stage III-IV.
