# Supplementary material for: Identification of a m6A-related ferroptosis signature as a potential predictive biomarker for lung adenocarcinoma
Source: BMC Pulm Med. 2023 Apr 18;23:128. doi: 10.1186/s12890-023-02410-x (PMC10111681; doi:10.1186/s12890-023-02410-x)
Supplement: Supplementary file 8 — Additional file 8: Figure S3. Differentiation of LUAD patients by PCA and t-SNE based on the risk model. [file 12890_2023_2410_MOESM8_ESM.docx]

**SUPPORTING INFORMATION**

**Figure S3. Differentiation of LUAD patients by PCA and t-SNE based on the risk model.**


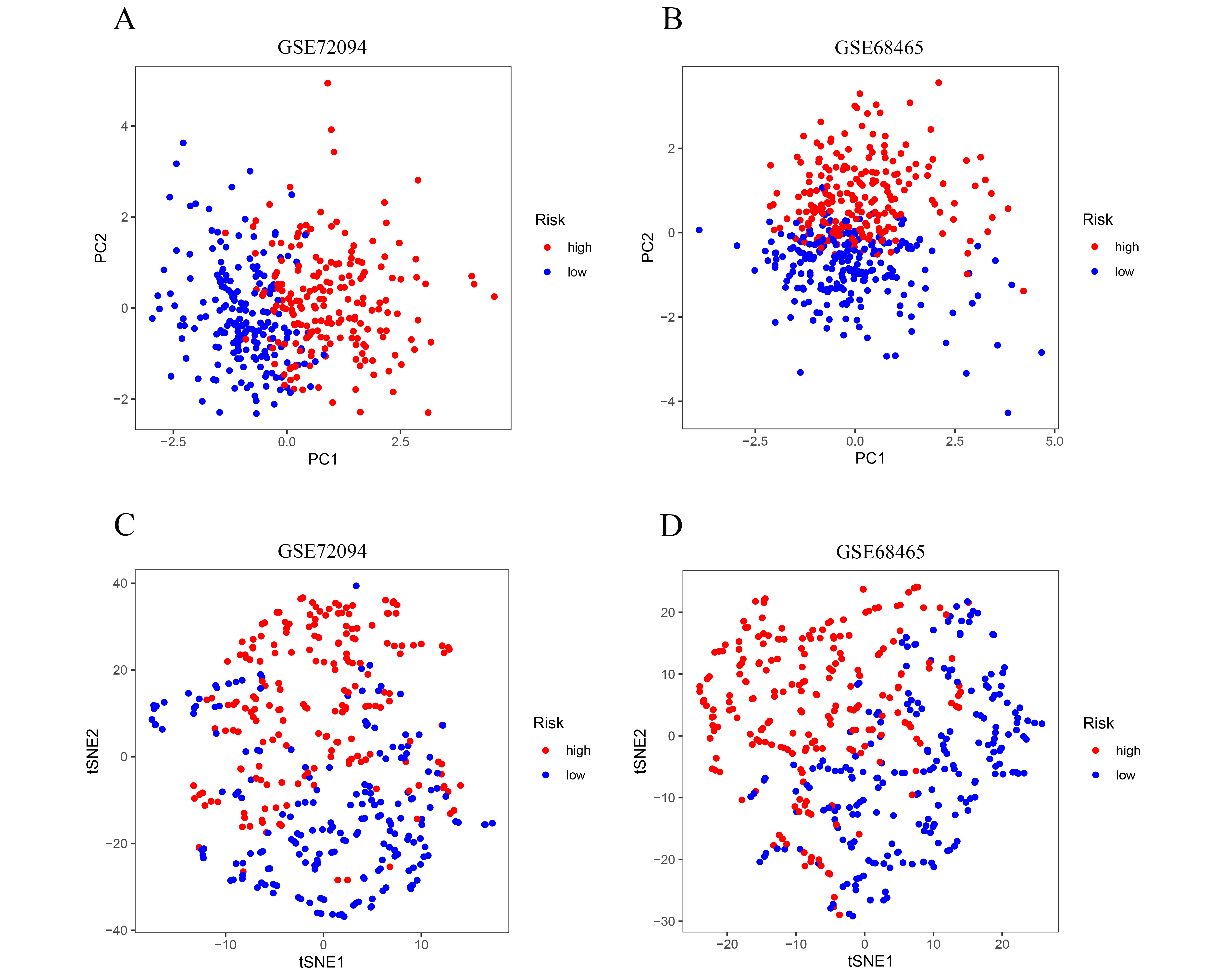


**Figure S3:** PCA and t-SNE of risk score in GEO databases. (A) PCA of GSE72094. (B) PCA of GSE68465. (C) t-SNE of GSE72094. (D) t-SNE of GSE68465.
